# Mental health status of the European population and its determinants: A cross-national comparison study

**DOI:** 10.1192/j.eurpsy.2025.2449

**Published:** 2025-04-24

**Authors:** Javier-David Lopez-Morinigo, Andrea Fiorillo, Geert Dom, Celso Arango

**Affiliations:** 1 Department of Child and Adolescent Psychiatry, Institute of Psychiatry and Mental Health, Hospital General Universitario Gregorio Marañón, IiSGM, Centro de Investigación Biomédica en Red de Salud Mental (CIBERSAM), School of Medicine, Universidad Complutense, Madrid, Spain; 2 Hospital Universitario del Sureste, Arganda del Rey, Madrid, Spain; 3 Department of Psychiatry, University of Campania “Luigi Vanvitelli,” Naples, Italy; 4 Collaborative Antwerp Psychiatric Research Institute (CAPRI), University of Antwerp, Antwerp, Belgium

**Keywords:** Europe, mental health determinants, mental health status, prevention, public health

## Abstract

**Background:**

This study aimed to provide an up-to-date cross-national comparison of the European population mental health (MH) *status* and its *determinants.*

**Methods:**

For the European Union (EU) 27 countries and the UK 6 Key Performance Indicators (KPIs) in *MH status* (e.g., prevalence of mental disorders) and 19 KPIs in individual (e.g., smoking), environmental (e.g., air pollution) and socioeconomic (e.g., poor housing conditions) *determinants of MH* were measured. KPIs scores were standardised in a 1–10 Likert Scale (1: worst performance; 10: best performance), thus allowing between-country comparisons of the relative performance. Exploratory unadjusted bivariate correlations between KPIs-transformed scores were run.

**Results:**

Based on the KPIs-transformed scores, Slovakia (8.3), Cyprus (7.8), and Greece (7.1) had the best MH status, while Sweden (3.1), UK (2.6), and The Netherlands (2.1) had the poorest MH status. Regarding determinants of MH Finland (8.0), Sweden, and Estonia (7.5) had the lowest MH risk, while France (3.1) and Romania (2.8) had the highest risk.

Smoking (*r* = −0.43, *p* = .021), alcohol use (*r* = 0.57, *p* = .002), daylight hours (*r* = 0.74, *p* < .001), ecoanxiety (*r* = −0.51, *p* = .005), air pollution (*r* = −0.46, *p* = .015), commuting time (*r* = 0.42, *p* = .026), and Fragile State Index (*r* = −0.44, *p* = .018) correlated with overall MH status.

**Conclusions:**

Population-level MH *status* and its *determinants* varied across European countries, including “low-risk, poor MH status” and “high-risk, good MH status” countries. Further non-tested determinants of MH and/or between-country differences in responsiveness to MH needs may explain this discrepancy. These results should guide future evidence-based public MH policymaking and universal preventive strategies in Europe.

## Introduction

The past few years have witnessed an unprecedented mental health (MH) crisis across the world [1, 2]. This challenging scenario has impeded progress towards achieving goals of global initiatives from the United Nations Sustainable Development Goal [3], the World Health Organization (WHO) [4], and the World Psychiatric Association (WPA) [5] aimed to promote MH and well-being.

Mental disorders have been linked to negative health and social outcomes [6]. Prior to the COVID-19 pandemic MH-related annual costs to society amounted to over EUR 600 billion (i.e., more than 4% of the Gross Domestic Product) across the 28 European Union (EU) countries, being the direct healthcare costs (EUR 190 billion) lower than the indirect costs due to unemployment and lost productivity (EUR 260 billion) [7]. Most importantly, patients with mental disorders [8], especially schizophrenia [9], were reported to have a 15–20-year shorter life expectancy than the general population. Of concern, most MH patients do not receive appropriate care [4, 10]; and the treatment gap [11] seems to have widened after the pandemic [12].

In the EU prior to the COVID-19 pandemic, 84 million (1 in 6 people) suffered from a mental disorder [13], which rose to almost 1 in 2 Europeans (46%) after this period [13], in spite of changes in MH services [14, 15]. The European population’s significant decline in MH has been largely attributed to the so-called *polycrisis*, that is, a perfect storm through the combination of adverse economic (e.g., economic recession), social (e.g., poor housing), geopolitical (e.g., Ukraine War) and environmental (e.g., climate change) risk factors of MH [16]. On the other hand, preventive psychiatry and public MH have increasingly gained traction over the past few years [17–20] and previous studies from our group identified some key modifiable MH risk factors [17, 21]. Hence, a better understanding of the population’s MH status and its determinants across European countries is critical for developing targeted preventive interventions aimed at improving Europeans’ MH status.

Within this context, the 2023 Headway Initiative (see below) collected and analyzed data on 54 MH-related key performance indicators (KPIs) across EU-27 countries and the UK. Based on these data, this study aimed to provide a cross-national comparison of the population MH *status* and its individual, environmental and social *determinants.*

## Methods

### The Headway Initiative

The Headway Initiative (hereafter, referred to as Headway) was launched by the Italian Think Tank The European House – Ambrosetti in partnership with Angelini Pharma in 2018, who also designed the Headway Mental Health Index 3.0 detailed below, which was presented to the European Parliament on 25 October 2023. In particular, by building upon the EU principle of “Health in All Policies,” the Headway project aimed to get new insights into the current MH status and its determinants across the 27 EU countries and the UK as well as their responsiveness in healthcare, workplaces, schools and society (Arango et al., this issue). Numerous multidisciplinary debates on social and health policies took place, which were led by more than 40 experts from the medical-scientific community and involved patient and family association representatives, health economists, and other relevant stakeholders [16].

**The 2023 Headway Mental Health Index 3.0: key performance indicators, variables, and data source**

The 2023 Headway Mental Health Index 3.0 [16] consists of 4 subindices and 54 KPIs in three domains: i) *Determinants* of MH (19 KPIs), ii) MH *status* of the population (6 KPIs), and iii) *Responsiveness* to MH needs in healthcare (14 KPIs), workplaces, schools, and society in general (15 KPIs). Some KPI included a set of variables selected through expert consensus meetings and data came from official open-access datasets. Tables 1 and 2 summarize the KPIs related to MH status and its determinants, respectively, including the variable(s) in each KPI, the measure, and data source, all of which were official, authoritative, and open-access datasets (e.g., Eurostat, OECD, WHO).

**Table 1.** Mental health status across Europe: The Headway Initiative methodology

KPI	Variable(s)	Measure	Data source
Prevalence	Prevalence of depression	Rate per 100,000 inhabitants	Global Burden of Disease, 2019
	Prevalence of anxiety		
	Prevalence of schizophrenia		
	Prevalence of bipolar disorder		
	Prevalence of ADHD*		
	Prevalence of CD*		
	Prevalence of LD*		
Incidence	Incidence of depression	Rate per 100,000 inhabitants	Global Burden of Disease, 2019
	Incidence of anxiety		
	Incidence of schizophrenia		
	Incidence of bipolar disorder		
	Incidence of ADHD*		
	Incidence of CD*		
	Incidence of LD*		
YLDs	YLDs for the general population	Rate per 100,000 inhabitants	Global Burden of Disease, 2019
	YLDs for under–20		
Mortality	MH-related mortality rates in under–20 in 202	Standardized rate per 100,000 inhabitants	Eurostat, 2021
Suicide	Suicide rates in 2020	Standardized rate per 100,000 inhabitants	Eurostat, 2020

KPI: Key Performance Indicator. YLDs: Years Lived with Disability. * Only for under-20 people.

URLs:

Global Burden of Disease, 2019: https://vizhub.healthdata.org/gbd-compare/.

Eurostat, 2021: https://ec.europa.eu/eurostat/databrowser/view/hlth_cd_aro__custom_11584303/default/table?lang=en.

Eurostat, 2020: https://ec.europa.eu/eurostat/databrowser/view/HLTH_CD_ASDR2__custom_11584510/default/table?lang=en.

**Table 2.** Determinants of mental health in Europe: the Headway Initiative methodology

KPI	Variable	Measure	Data source
*Individual factors*			
Smoking	Daily smoking	% population	OECD. Health at a Glance, 2022.
Alcohol abuse	Heavy episodic drinking in the last month	% population	OECD. Health at a Glance, 2022.
Drugs abuse	Use of illicit drugs in the last year	% population	European Drug Report 2022
Bullying	Weekly bullying	% school population	PISA, 2018
Sexual Abuse	YLD rate per 100,000 population	% school population	Global Burden of Disease, 2021
*Environmental factors*			
Hours of daylight	Annual hours of daylight in European capitals	Number of hours	National databases, 2020
Temperature increase	Average Temperature increase: 2022 vs. 1951–1980		IMF, 2022
Extreme weather impact	Loss per capita due to extreme weather 1980–2021	Euros per capita	European Environment Agency, 2022
Natural disasters	Risk of natural disasters	%	EHS, 2022
Ecoanxiety	Exposure climate change-related threats	% population	Eurobarometer, 2023
Air pollution	Exposure to PM2,5 in urban areas	% population	EEA, 2022
Noise pollution	Feel impacted by noise, 2020	% population	Eurostat, 2021
Transport and road traffic	Hours per year	Traffic index	TomTom, 2019
Urban Green Space	Green infrastructure over total area	%	European Environment Agency, 2021
Commuting time	People taking over 30 minutes to work, 2019	% population	Eurostat, 2019
*Socio-economic factors*			
Fragile State Index	Score	Score	Fund for peace, 2023
Poor Housing	People living in poor housing conditions	% population	Eurostat, 2021
Overcrowding rate	People living in overcrowded houses	% population	Eurostat, 2022
Crime level	Weighted average of crime indicators	Crime Index	Eurostat, 2020

URLs:

OECD. Health at Glance, 2022: https://www.oecd-ilibrary.org/social-issues-migration-health/daily-smoking-rates-among-adults-by-gender-2020-or-nearest-year_aade1255-en.

European Drug Report 2022: https://www.euda.europa.eu/publications/edr/trends-developments/2022_en.

PISA, 2018: https://www.oecd-ilibrary.org/education/pisa-2018-results-volume-iii_acd78851-en.

Global Burden of Disease, 2021: https://vizhub.healthdata.org/gbd-compare/.

IMF, 2022: https://climatedata.imf.org/pages/climatechange-data.

European Environment Agency, 2022: https://www.eea.europa.eu/en/analysis/indicators/economic-losses-from-climate-related#:~:text=In%20absolute%20terms%2C%20the%20highest,in%20Belgium%2C%20Germany%20and%20Luxembourg.

EHS, 2022: Institute for Environment and Human Security: https://en.wikipedia.org/wiki/List_of_countries_by_natural_disaster_risk.

Eurobarometer, 2023: https://europa.eu/eurobarometer/surveys/detail/2954.

EEA, 2022: European Environment Agency: https://www.eea.europa.eu/publications/status-of-air-quality-in-Europe-2022/europes-air-quality-status-2022.

Eurostat, 2021: https://data.europa.eu/data/datasets/fxzwh5qqu5iplmua0m5tq?locale=it.

TomTom, 2019: https://www.numbeo.com/traffic/region_rankings.jsp?title=2023&region=150.

Fund for peace, 2023: https://en.wikipedia.org/wiki/List_of_countries_by_Fragile_States_Index.

Eurostat, 2021: https://ec.europa.eu/eurostat/databrowser/view/tessi292/default/table?lang=en&category=t_ilc.t_ilc_md.t_ilc_mdho.

Eurostat, 2022: https://ec.europa.eu/eurostat/databrowser/view/tessi170/default/table?lang=en&category=t_ilc.t_ilc_lv.t_ilc_lvho.t_ilc_lvho_or.

Eurostat, 2020: https://ec.europa.eu/eurostat/databrowser/view/crim_off_cat/default/table?lang=en&category=crim.crim_off.

First, for each variable, a maximum score (10) and a minimum score (1) were assigned to the best and worst-performing countries, respectively. Second, for each country with an intermediate performance, a score ranging from 1 to 10 was assigned as detailed below, thus making the *relative* performance of each country comparable across the board.

scale = (best performer − worst performer)/(max score − min score).

score = [(value of Countryi − worst performer)/scale + 1].

When the KPI was composed of multiple sub-indicators (or variables), the score was assigned to each sub-indicator. The final score was calculated as the average of the scores on the sub-indicators. After calculating the score for each KPI, a score was assigned for each area based on the average of the KPI scores, weighted by the assigned weights. For MH status KPIs higher scores indicated “better” MH (e.g., lower prevalence of mental disorders or suicide rates). Regarding determinants of MH higher KPI scores indicated lower risk (e.g., lower prevalence of alcohol use) and vice versa.

Six KPIs in MH *status* included i) *prevalence* and ii) *incidence* of depression, autism spectrum disorders, anxiety, schizophrenia, bipolar disorder and, only for under-20 individuals, attention-deficit hyperactivity disorder (ADHD), conduct disorder (CD) and learning disability (LD); iii) *years lived with disability* (YLDs) for the general population and iv) for under-20 individuals, v) MH-related *mortality* and vi) *suicide* rates (Table 1).

Nineteen KPIs in *determinants* of MH encompassed 5 individual (smoking, alcohol, drugs use, sexual abuse, and bullying), 10 environmental (hours of daylight, temperature increase, economic damage by extreme weather events, natural disasters, ecoanxiety, air and noise pollution, transport and road traffic, urban green space, and commuting time) and 4 socioeconomic factors (Fragile State Index, poor housing, overcrowding rate, and crime level) (Table 2).

**Figure 1.**
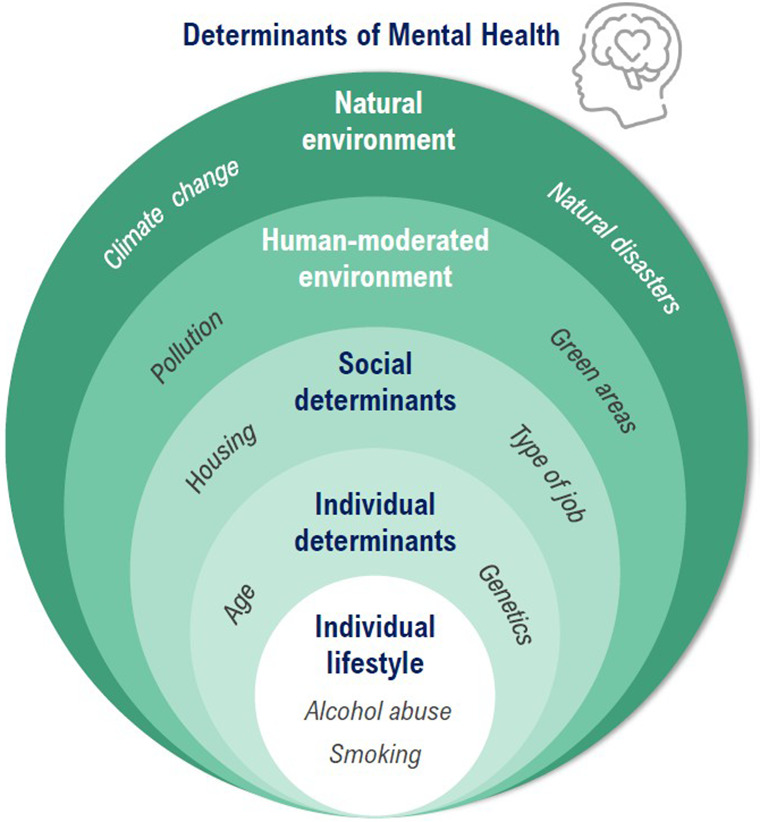
Interrelationships among individual, environmental and socieconomic determinants of mental health.

Figure 1 shows the interrelationships among individual, environmental, and socioeconomic determinants of MH.

### Statistics

For descriptive purposes, all KPIs scores of the EU-27 + UK countries ordered alphabetically were reported. Bivariate correlations explored potential associations between KPI scores, which were reported as Pearson coefficients and the corresponding p-value since all Headway-transformed KPI scores, which ranged from 1 to 10, followed a normal distribution. Given the exploratory nature, these analyses were not corrected for multiple testing or adjusted for potential confounders. For all the above analyses, which were performed with the Statistical Package for Social Science version 25.0 (SPSS Inc., Chicago, IL, USA), a two-tailed significance level was set at *p* < .05.

## Results

### Mental health status across European countries

MH status KPI scores are detailed in Table 3. Overall MH status scores showed Slovakia (8.3), Cyprus (7.8), and Greece (7.1) to achieve the best performance, while The Netherlands (2.1), the UK (2.6), and Sweden (3.1) had the poorest MH status.

**Table 3.** Mental health status across European countries: Headway-transformed KPI scores

	Prevalence	Incidence	YLDs general	YLDs < 20	Mortality	Suicide	TOTAL score
Austria	6,7	5,5	6,1	5,1	6,3	5,8	5,1
Belgium	7,6	4,8	8,2	9,9	5,4	4,0	4,1
Bulgaria	8,7	10,0	4,0	2,8	5,9	7,7	6,3
Croatia	7,4	9,1	9,8	10,0	7,1	5,1	5,8
Cyprus	7,2	4,1	7,5	9,5	6,8	10,0	7,8
Czechia	9,0	9,1	2,2	1,0	8,8	5,9	5,8
Denmark	8,0	6,1	6,4	6,2	4,0	6,7	4,5
Estonia	7,8	7,8	8,9	9,7	9,4	3,5	5,7
Finland	5,1	5,7	5,7	5,4	4,4	5,2	4,1
France	5,6	3,7	4,4	4,7	6,6	4,9	4,2
Germany	7,5	7,1	5,3	5,5	2,4	6,5	4,7
Greece	2,6	5,1	2,3	3,9	8,0	9,7	7,1
Hungary	8,1	9,3	10,0	9,8	5,8	3,1	4,3
Ireland	1,7	1,0	3,4	4,2	6,8	6,9	3,2
Italy	3,0	6,1	4,8	5,2	5,6	8,9	6,4
Latvia	8,0	8,0	8,7	9,6	6,0	3,9	6,0
Lithuania	6,5	7,8	7,8	9,1	9,2	1,0	3,8
Luxembourg	4,2	4,7	5,8	5,8	5,5	6,7	4,6
Malta	6,0	5,9	5,4	5,3	3,5	9,7	6,4
Netherlands	2,5	3,7	4,0	4,6	3,0	6,4	2,1
Poland	7,6	8,8	8,2	8,6	5,9	5,7	5,6
Portugal	1,0	5,1	1,0	2,4	3,6	7,5	4,3
Romania	7,8	8,6	6,9	7,7	5,9	7,2	6,3
Slovakia	10,0	8,7	7,0	8,2	10,0	8,3	8,3
Slovenia	9,0	9,1	9,7	10,0	8,6	3,1	5,7
Spain	1,1	4,2	2,6	3,4	5,9	7,8	4,5
Sweden	3,5	3,5	5,2	5,8	3,5	5,7	3,1
United Kingdom	5,2	3,1	5,5	6,0	1,0	6,4	2,6

The raw data on MH status variables for the above KPIs are provided in the online supplementary material, namely the prevalence (Supplementary Table S1) and incidence (Supplementary Table S2) of the above mental disorders and MH-related mortality and suicide rates (Supplementary Table S3).

### Determinants of mental health across European countries

Headway-transformed scores on KPIs in individual, environmental, and socioeconomic determinants of MH are detailed in Table 4.

**Table 4.** Individual, environmental and socioeconomic determinants of mental health across European countries: Headway Initiative Index scores

	Individual	Environmental	Socioeconomic	Determinants	Status
Austria	3,2	6,0	7,2	5,5	5,1
Belgium	5,3	4,5	6,5	5,4	4,1
Bulgaria	2,4	5,9	3,2	3,9	6,3
Croatia	3,7	5,2	2,3	3,7	5,8
Cyprus	9,2	9,6	2,6	7,1	7,8
Czechia	4,3	5,3	5,7	5,1	5,8
Denmark	1,8	5,9	7,4	5,0	4,5
Estonia	6,9	10,0	5,8	7,5	5,7
Finland	6,4	9,6	8,1	8,0	4,1
France	3,1	2,4	3,9	3,1	4,2
Germany	1,0	4,9	7,6	4,5	4,7
Greece	6,8	1,0	2,0	3,3	7,1
Hungary	5,5	4,1	3,6	4,4	4,3
Ireland	5,3	6,1	8,3	6,6	3,2
Italy	4,5	3,7	3,6	3,9	6,4
Latvia	4,8	5,8	1,0	3,9	6,0
Lithuania	5,5	8,1	4,0	5,9	3,8
Luxembourg	4,8	4,9	7,5	5,7	4,6
Malta	7,7	5,3	8,7	7,2	6,4
Netherlands	3,5	6,2	10,0	6,6	2,1
Poland	7,2	5,0	2,9	5,1	5,6
Portugal	6,5	5,0	6,0	5,8	4,3
Romania	2,6	2,3	3,5	2,8	6,3
Slovakia	5,2	6,2	3,3	4,9	8,3
Slovenia	6,4	5,9	5,9	6,1	5,7
Spain	5,1	4,3	3,8	4,4	4,5
Sweden	10,0	7,5	5,1	7,5	3,1
United Kingdom	3,9	4,2	2,5	3,5	2,6

#### Individual determinants

Sweeden (10), Cyprus (9.2), and Malta (7.7) were the lowest MH risk countries, whereas Germany (1.0), Denmark (1.8), and Bulgaria (2.4) had the highest risk. In the online supplementary material, we have provided the raw data (Supplementary Table S4) and the Headway-transformed KPI scores (Supplementary Table S5).

#### Environmental determinants

Estonia (10), Cyprus, and Finland (9.6) had the lowest MH risk, whereas Greece (1.0), Romania (2.3), and France (2.4) had the highest risk. The full raw data (Supplementary Table S6) and Headway-transformed KPI scores (Supplementary Table S7) are available in the online supplementary material.

#### Socioeconomic determinants

The Netherlands (10), Malta (8.4), and Ireland (8.3) had the lowest MH risk, while Latvia (1.0), Greece (2.0), and Croatia (2.3) had the highest risk. See the online supplementary material for further details of the raw data (Supplementary Table S8) and Headway-transformed KPI scores (Supplementary Table S9).

#### Overall scores

Overall, Finland (8.0), Estonia (7.5), and Sweeden (7.5) showed the most favourable determinants of MH (i.e., the lowest MH risk), while Romania (2.8), France (3.1) and Greece (3.3) had the highest MH risk.

### Relationship between status and determinants of mental health

In Table 4 we have also added data on overall status KPIs scores (right column). By using a traffic light colours system and comparing the colour in determinants and status KPIs global scores five clusters of countries were found as follows: i) “Red-Red” - ‘High risk, poor MH’: the UK; ii) “Green-Green” - ‘Low risk, good MH: Cyprus, Malta; iii) “Red-Green” - ‘High risk, good MH’: Bulgaria, Croatia, Romania, Greece, Italy, Latvia; iv) “Green-Red” – ‘Low risk, poor MH’: Ireland, Lithuania, The Netherlands, Sweden, Hungary; and v) “Any Yellow” – ‘Medium risk, average MH’: Austria, Belgium, Czechia, Denmark, Estonia, Finland, France, Germany, Luxembourg, Poland, Portugal, Slovenia, Slovakia, Spain.

At an exploratory level, we ran unadjusted bivariate correlations between determinants and status global KPIs scores, which are detailed in Table 5. Smoking (*r* = −0.43, *p* = .021), alcohol (*r* = 0.57, *p* = .002), hours of daylight (*r* = 0.73, *p* < .001), ecoanxiety (*r* = −0.51, *p* = .005), air pollution (*r* = −0.46, *p* = .015), commuting time (*r* = 0.42, *p* = .026) and Fragile State Index (*r* = −0.44, *p* = .018) correlated with an overall measure of MH status.

**Table 5.** Relationship between determinants and status KPIs scores across European countries

	Prevalence		Incidence		YLD_AD		YLD_Child		Mortality		Suicide		Status	
	r	p	r	p	r	p	r	p	r	p	r	p	r	p
*Individual factors*	−0.21	.285	−0.19	.327	−0.05	.779	0.22	.260	0.17	.397	0.08	.700	0.21	.292
Smoking	−0.29	.127	**−0.38**	**.045**	−0.15	.450	−0.08	.689	−0.39	.039	−0.15	.450	**−0.43**	**.021**
Alcohol abuse	−0.17	.382	0.09	.660	−0.19	.325	−0.01	.961	0.37	.049	0.27	.165	**0.57**	**.002**
Drugs abuse	0.27	.170	**0.42**	**.024**	.121	.539	0.24	.224	0.01	.622	0.05	.789	0.29	.128
Bullying	**−0.38**	**.046**	**−0.37**	**.050**	−0.21	.271	−0.10	.65	−0.15	.439	−0.14	.148	−0.27	.156
Sexual abuse	**0.67**	**<.001**	**0.51**	**.006**	**0.72**	**<.001**	**0.74**	**<.001**	**0.55**	**.003**	**−0.42**	**.026**	−0.10	.596
*Environmental factors*	0.18	.352	.01	.944	0.28	.156	0.272	.161	0.14	.463	−0.25	.193	−0.20	.323
Hours of daylight	0.24	.218	−0.04	.853	−0.34	.081	0.13	.504	0.01	.960	**0.66**	**<.001**	**0.73**	**<.001**
Temperature increase	0.04	.835	.011	.955	0.03	.858	0.16	.410	0.05	.783	0.28	.156	0.22	.251
Weather events	0.16	.409	0.17	.393	0.23	.237	0.33	.086	0.10	.595	−0.03	.880	−0.01	.991
Natural disasters	**0.58**	**.001**	0.33	.089	**0.51**	**.005**	0.26	.183	0.14	.485	−0.32	.098	−0.30	.134
Ecoanxiety	0.01	.615	−0.15	.448	0.14	.466	−0.01	.953	0.10	.595	−0.48	.010	**−0.51**	**.005**
Air pollution	**−0.37**	**.050**	**−0.59**	**.001**	−0.20	.315	−0.14	.450	−0.34	.073	−0.27	.161	**−0.46**	**.015**
Noise pollution	**0.51**	**.006**	**0.48**	**.010**	**.55**	**.003**	**0.46**	**.015**	**0.59**	**.001**	**−0.38**	**.049**	−0.06	.757
Transport and traffic	0.24	.213	0.23	.234	0.24	.231	0.03	.874	0.12	.53	−0.22	.257	−0.14	.467
Urban green space	**0.40**	**.034**	**0.46**	**.014**	**0.54**	**.003**	**0.46**	**.015**	0.36	.061	**−0.61**	**.001**	−0.27	.162
Commuting time	−026	.174	−0.11	.578	−0.35	.065	−0.09	.638	0.28	.153	0.26	.189	**0.42**	**.026**
*Socio-economic factors*	−0.23	.238	−0.35	.067	−0.14	.470	−0.31	.111	−0.32	.098	−0.01	.969	−0.31	0.110
Fragile State Index	−0.28	.154	**−0.44**	**.018**	−0.13	.508	−0.25	.200	**−0.40**	**.035**	−0.19	.333	**−0.44**	**.018**
Poor Housing	0.22	.261	0.31	.110	0.16	.426	−0.13	.51	0.06	.749	−0.11	.560	−0.08	.689
Overcrowding rate	**−0.39**	**.039**	−0.68	<.001	−0.28	.148	−0.25	.199	**−0.39**	**.042**	0.10	.613	−0.25	.193
Crime level	0.25	.199	0.55	.002	0.15	.446	0.15	.434	**0.56**	**.002**	0.08	.679	0.39	.038
Determinants	−0.13	.492	−0.27	.167	0.03	.892	0.07	.738	−0.02	.924	−0.09	.647	−0.16	.419

In bold, statistically significant (p < .05) correlations.

## Discussion

### Principal findings

This first Headway-based study aimed to carry out a comparison of the population MH *status* and its *determinants* across European countries. Two main findings emerged from the analyses. First, as expected, there were relevant differences in the population MH status and its determinants across European countries. Second, somehow surprising, for up to 11 countries MH status differed from what a determinants-based risk assessment appeared to suggest, including both “low risk, poor MH status”, and “high risk, good MH status” countries. Thus, Slovakia and Cyprus emerged as the “healthiest” countries, whereas Finland, Sweden, and Estonia were the lowest-risk countries according to the MH determinants KPIs. In brief, between-country differences in their responsiveness to the population MH needs may, in large part, explain this discrepancy, which forms the basis for the second Headway article in this issue.

### Mental health status of the European population

A deeper theoretical debate about the conceptualization of MH, although still warranted [22, 23], falls outside the scope of this article. This noted, Slovakia, Cyprus, and Greece emerged as the countries with the best population MH status, whereas Sweden, the UK, and The Netherlands had the poorest MH status. However, much caution is needed when interpreting these results, which take into account multiple variables and may have been affected by misreporting issues. Specifically, it is worth noting that those countries with worse MH status could just reflect better quality of data/reporting, which appears to apply to those countries with greater MH expenditure, such as Scandinavian countries.

Of concern, the COVID-19 pandemic triggered a 25% increase in the prevalence of anxiety and depressive disorders [4], with a prevalence of anxiety ranging from 4.3% (Estonia) to 11.3% (Portugal) and a prevalence of depression ranging from 4.2% (Slovakia) to 7.5% (Spain). The prevalence of schizophrenia, however, was shown to have a much smaller variation (0.5% in The Netherlands vs. 0.3% in Denmark) compared with between-country differences in the incidence of first-episode psychosis [24].

Over the past three decades, data from the Global Burden of Disease project have replicated mental disorders to be a major contributor to disability [6]. In line with this, our results showed a widely used measure of disability, namely the number of years lived with disability (YLD), to range from 1617.8 (Hungary) to 2603.9 (Portugal), hence unacceptably high across Europe, which will require coordinated delivery of effective prevention and treatment programmes by governments and the global health community.

More importantly, mortality, particularly suicide, can be considered as the most tragic outcome in MH. In this respect, mortality rates per 100,000 inhabitants in 2021 ranged from 8.9 (Slovakia) to 84.0 (UK), i.e., an almost 10-fold variation, which may have been due to reporting differences, especially for deaths by natural causes, which cannot be easily linked with MH issues by the relevant authority across countries. This is less likely to apply to suicide rates (per 100,000 inhabitants) which in 2020 ranged from 3.45 (Cyprus) to 21.25 (Lithuania), hence a 6-fold variation. Both mortality and suicide rates have remained unchanged in Europe for the past few years, as supported by the Global Burden of Disease 2019 study [25]. Addressing the mortality gap in MH, particularly in schizophrenia [9], through psychosocial interventions targeting modifiable risk factors, such as unhealthy lifestyles [26], urges multi-agency action worldwide [27, 28].

### Determinants of mental health of the European population

The well-established individual, environmental, and social determinants of MH have been demonstrated to be unequally distributed within- and between populations [4, 29], which is in full agreement with our data showing high variation across the board. This raises a fundamental question: to what extent is one’s MH (pre)determined by external socioeconomic and environmental factors? Truly, up to 62% of Europeans, especially women (67%), were affected by the aforementioned post-pandemic polycrisis [13]. In keeping with this, the 2023 Headway Mental Health Index 3.0 incorporated some relatively novel polycrisis-related KPIs, such as the impact of natural disasters, eco-anxiety, and crime level on MH [16].

The effects of *natural disasters* on MH are well-established [30]. In Europe the number of natural disasters has grown from 91 in 1979 to 1,452 in 2019, accounting for over 145,000 deaths over the past 40 years [16]. Although exposure to these events has been linked with negative MH outcomes [30], adequate MH support, community resilience initiatives, and disaster preparedness measures [31], including the use of mobile apps [32], may mitigate this.

*Eco-anxiety*, which can be defined as a” pre-traumatic stress disorder in response to climate change and ecological crises,” has become a focus of major concern in global public MH [33], including Europe [34]. Eco-anxiety appears to particularly affect children and adolescents’ MH [35] irrespective of neuroticism and/or personal beliefs [36]. Combating climate change may therefore contribute to preventing mental disorders via reduced risk of eco-anxiety, especially for youth, a universal prevention measure to which the European Psychiatric Association (EPA) is particularly committed [34].

*Crime level* has been long associated with poorer MH and identified as a barrier to engagement in health-promoting activities [37]. Truly, community violence was linked with poorer MH outcomes [38]. While victims of crime may benefit from targeted prevention interventions, future studies should clarify the extent to which crime level affects MH, while controlling for social disadvantage and related factors.

### Relationship between mental health status and its determinants across Europe

As noted above (Table 4), the extent to which determinants of MH predicted the population MH status across countries was found to be somehow weak. In particular, there were both “high risk, good MH” countries, such as Bulgaria, Croatia, Romania, Greece, Italy, and Latvia; and “low risk, poor MH” countries, namely Ireland, Lithuania, The Netherlands, Sweden, and Hungary. Although further non-tested risk/protective factors may contribute to MH and quality of data and reporting issues should be considered, this is likely to be explained, in large part, by countries’ responsiveness to their citizens’ MH needs (see Arango et al., this issue).

Based on the bivariate associations between KPI scores (Table 5), alcohol use, smoking, hours of daylight, commuting time, ecoanxiety, air pollution, and Fragile State Index emerged as the *common* determinants of MH across Europe. *Smoking and alcohol use* have been consistently linked to poorer (mental) health outcomes worldwide [39] in spite of significant progress in fighting both addictions [40]. Daylight exposure was linked with better MH [41], consistent with our data showing a positive relationship between more hours of daylight and lower suicide rates [42]. Interestingly, ecoanxiety can be both cause and consequence of mental disorders [33]. Commuting time, which can be defined as the proportion of people who take over 30 minutes to go to work, showed a positive correlation with MH status – the lower the proportion of people with long commuting times, the better the MH. Reducing commuting time may therefore prevent mental disorders, such as depression in adults [43] and in adolescents [44], which warrants future intervention studies. The Fragile State Index can be considered as a proxy measure of social cohesion, economic status and political stability of countries, which is inextrincably linked to most determinants of MH [29], thus behaving as a major MH risk factor, especially for child maltreatment [45]. Poverty alleviation programmes, which have been recommended by the Lancet-Commission [46], may reduce this risk [47].

Also, sexual abuse, noise pollution, and the proportion of green areas correlated with most status KPIs except mortality and suicide (Table 5). Between 11% (men) and 13% (women) of MH service users were meta-analytically found to have suffered from sexual abuse, a well-established predictor of poor MH outcomes [48–50]. Although noise pollution was thought to worsen MH, high-quality longitudinal studies showing the benefits of noise-reducing policies are lacking [51]. Finally, our findings revealed that countries with a higher proportion of green area space achieved better MH status, except for suicide outcomes, which was probably due to the link between rurality and increased suicide risk, hence, a complex issue worthy of further investigation [52].

### Next steps

This first Headway data-based study provided an overview of the population MH status and its determinants across Europe, which may pave the way towards more targeted prevention interventions. In short, this study may provide new insights into the extent to which one’s MH status is determined by such a complex interplay of individual, environmental, and social factors, which is not to underestimate the role of genetics and other neurobiological variables in the aetiology and outcomes of mental disorders [21].

Interestingly, a well-studied indicated prevention intervention in psychiatry, such as the “At clinical high-risk for psychosis” (CHR-P) model [53], has largely failed to predict [54] and prevent [55] transition to psychosis. Even in a catchment area with well-resourced CHR-P clinics, such as South-East London (UK), only 4.1% of first-episode psychosis incident cases had presented to these CHR-P clinics and met CHR-P criteria [56]; hence, of little value from a public health and/or economic perspective [57]. Alternatively, from a universal prevention approach, decreasing population exposure to well-known risk factors for psychosis, such as cannabis use [58], may be more effective [59]. Consistent with this public health model, some evidence-based prevention measures in MH can be recommended [17], which were also demonstrated to be cost-effective, especially in children and adolescents [60, 61]. Of note, most major mental disorders onset occurs before age 25 [62], thus making childhood and adolescence the optimal period to deliver any preventive intervention [19]. In line with this notion, Table 6 provides some examples of primary prevention strategies targeting the common determinants of MH across Europe.

**Table 6.** Proposed prevention strategies targeting the determinants of mental health in Europe

Determinants	Intervention	Type of prevention	Level of evidence
*Individual factors*			
Smoking, alcohol and drugs	School-based SEL	Universal Primary	MA [65]
	Digital Mental Health	Universal Primary	MA [68]
	School-based programmes	Universal Primary	MA [72]
Bullying	School-based Interventions	Universal Primary	MA [73]
Sexual abuse	School-based Intervention	Selected Primary	MA [74]
*Environmental factors*			
Natural disasters	Mobile apps	Universal Primary	MA [32]
Ecoanxiety	Planetary health education	Universal Primary	Future research needed
Air pollution	Nature-based Interventions	Universal Primary	Future research needed
Noise pollution	Nature-based Interventions	Universal Primary	Future research needed
Green space	Nature-based Interventions	Universal Primary	Future research needed
Commuting time	Nature-based Interventions	Universal Primary	Future research needed
*Socioeconomic factors*			
Fragile State Index	Poverty alleviation programmes	Universal Primary	MA [47]

Abbreviations: SEL: social and emotional learning. MA: meta-analysis.

Prevention in MH has attempted to reduce exposure to well-established *risk* factors thus far. However, in the years to come the focus should be switched towards promoting *protective* factors [19], such as resilience [63], physical activity [64], school-based social, and emotional learning [65]. Of note, this MH promotion model has proved useful in asylum seekers children and adolescents [66], which is in line with global strategies promoting physical health in psychiatry [20] and the 2023 European Commission (EC) Mental Health Strategy [67]. More controversially, the long-term benefits of new technologies, such as smartphone-based apps, remain less clear and their recommendation, particularly to the youth, raises ethical issues [68].

Advancing in preventive psychiatry, however, seems to be hampered by stigma [69], which underestimates the general public perception of the need for MH prevention, and financial issues. In particular, its *long-term* high return appears to discourage health authorities and policymakers from investing in MH prevention as a priority [17].

### Strengths and limitations

Data supporting this study’s findings came from the updated 2023 Headway Mental Health Index 3.0, which measured 54 MH-related KPIs across EU-27 countries and the UK, thus allowing direct country-to-country comparisons. All data sources were official authoritative open-access datasets. Results may therefore inform some evidence-based public MH strategies and universal primary prevention interventions.

However, this study has three limitations. First, the Headway methodology relied partly on national datasets which differed in quality of data, which also were collected during different years. Specifically, mis- and under-reporting issues should be considered. Second, non-tested KPIs, such as mass media use or fear of war, may influence the European population MH. Third, both analytical and qualitative approaches were adopted, which may have incorporated some biases, although this seems unlikely.

### Final remarks

The post-pandemic polycrisis [16] has put MH at the top of the political agenda of numerous institutions and governments, including the European Commission [67]. This is therefore a unique opportunity to implement a new roadmap for MH in Europe [16] under the scientific leadership of the EPA, although the challenges ahead will require increased efforts. In particular, while precision psychiatry cannot yet inform clinical decision-making at an individual level [70], *universal preventive psychiatry* seems to be much more within our grasp [71].

## Supporting information

10.1192/j.eurpsy.2025.2449.sm001 Lopez-Morinigo et al. supplementary material Lopez-Morinigo et al. supplementary material

## Data Availability

All the data supporting the findings of this study are available in the online supplementary material.

## Supplementary material

The supplementary material for this article can be found at http://doi.org/10.1192/j.eurpsy.2025.2449.
