# Supplementary material for: Mental health status of the European population and its determinants: A cross-national comparison study
Source: Eur Psychiatry. 2025 Apr 24;68(1):e103. doi: 10.1192/j.eurpsy.2025.2449 (PMC12344470; doi:10.1192/j.eurpsy.2025.2449)
Supplement: Lopez-Morinigo et al. supplementary material [file S0924933825024496sup001.zip › Table_S1_Prevalence_Results_Raw.doc]

| **Table S1. Raw data on the prevalence of mental disorders (rate per 100,000 inhabitants)** | | | | | | | | |
| --- | --- | --- | --- | --- | --- | --- | --- | --- |
|  | **Depression** | **Autism** | **Anxiety** | **Schizoph.** | **Bipolar disorder** | **ADHD** | **Conduct disorders** | **Intellectual disorders** |
| Austria | 4642,853 | 526,0 | 7746,682 | 345,3 | 624,0 | 2872,1 | 1711,3 | 359,2 |
| Belgium | 5626,314 | 526,1 | 6855,442 | 328,0 | 594,0 | 1746,0 | 1563,9 | 764,0 |
| Bulgaria | 4740,937 | 335,6 | 4958,765 | 354,9 | 622,3 | 2347,3 | 1778,8 | 384,8 |
| Croatia | 4790,167 | 346,8 | 5099,4 | 356,7 | 1041,5 | 1819,7 | 1677,7 | 603,0 |
| Cyprus | 4357,154 | 535,3 | 8227,031 | 344,1 | 619,8 | 1848,6 | 1712,7 | 469,4 |
| Czechia | 4537,294 | 350,9 | 4502,117 | 364,7 | 636,2 | 2289,2 | 1716,9 | 457,4 |
| Denmark | 4786,672 | 403,8 | 5886,887 | 286,6 | 1050,2 | 1698,1 | 1815,3 | 78,2 |
| Estonia | 5604,289 | 369,5 | 4297,364 | 344,1 | 1007,1 | 1793,0 | 1640,0 | 466,7 |
| Finland | 5291,288 | 514,7 | 4634,281 | 315,7 | 1060,3 | 3002,7 | 1829,9 | 494,7 |
| France | 5887,301 | 425,4 | 8835,393 | 311,6 | 615,6 | 3018,6 | 1819,8 | 510,4 |
| Germany | 4748,92 | 525,3 | 7773,552 | 325,6 | 713,8 | 1202,4 | 1768,9 | 305,8 |
| Greece | 7358,461 | 515,7 | 7327,468 | 338,6 | 1057,0 | 2384,3 | 1838,5 | 490,7 |
| Hungary | 4300,397 | 340,3 | 4496,804 | 358,9 | 1059,7 | 1831,4 | 1704,5 | 443,3 |
| Ireland | 5913,976 | 583,7 | 9021,024 | 408,6 | 1080,9 | 2966,3 | 1783,7 | 329,3 |
| Italy | 5513,31 | 516,1 | 7324,654 | 368,7 | 1040,4 | 2203,3 | 1963,7 | 454,8 |
| Latvia | 5829,252 | 362,5 | 5008,092 | 340,4 | 888,8 | 1764,7 | 1614,3 | 523,3 |
| Lithuania | 6621,255 | 369,3 | 6234,937 | 343,2 | 1029,0 | 1766,3 | 1603,8 | 468,9 |
| Luxembourg | 5006,722 | 541,7 | 7256,491 | 354,2 | 1213,0 | 2341,0 | 1775,4 | 249,4 |
| Malta | 4421,604 | 538,0 | 8151,064 | 343,0 | 863,1 | 2297,1 | 1731,0 | 382,8 |
| Netherlands | 5294,152 | 591,5 | 9165,142 | 448,4 | 1012,4 | 2714,3 | 1720,9 | 232,4 |
| Poland | 4562,663 | 448,7 | 5597,547 | 364,5 | 697,6 | 1869,6 | 1764,3 | 494,3 |
| Portugal | 7421,378 | 496,6 | 11320,38 | 338,6 | 1049,0 | 2419,3 | 1891,5 | 488,9 |
| Romania | 4449,128 | 335,9 | 4985,191 | 350,1 | 1001,6 | 1843,4 | 1721,6 | 558,6 |
| Slovakia | 4217,895 | 349,2 | 4760,525 | 369,0 | 627,2 | 1779,1 | 1627,4 | 458,6 |
| Slovenia | 4942,065 | 351,4 | 4880,791 | 367,6 | 766,4 | 1789,9 | 1619,4 | 397,9 |
| Spain | 7548,722 | 535,1 | 6695,518 | 333,6 | 585,5 | 4927,9 | 1846,0 | 1448,3 |
| Sweden | 5836,793 | 661,9 | 6143,839 | 326,3 | 1011,2 | 1927,9 | 1859,8 | 613,9 |
| UK | 6017,598 | 700,1 | 5964,281 | 293,0 | 587,3 | 2414,5 | 1905,3 | 366,6 |
