# Supplementary material for: Mental health status of the European population and its determinants: A cross-national comparison study
Source: Eur Psychiatry. 2025 Apr 24;68(1):e103. doi: 10.1192/j.eurpsy.2025.2449 (PMC12344470; doi:10.1192/j.eurpsy.2025.2449)
Supplement: Lopez-Morinigo et al. supplementary material [file S0924933825024496sup001.zip › Table_S2_Incidence_Results_Raw.doc]

| **Table S2. Raw data on incidence of mental disorders per 100,000 inhabitants** | | | | | | | | |
| --- | --- | --- | --- | --- | --- | --- | --- | --- |
|  | **Depression** | **Autism** | **Anxiety** | **Schizoph.** | **Bipolar disorder** | **ADHD** | **Conduct disorders** | **Intellectual disorders** |
| Austria | 3825 | 6,6 | 736 | 15,3 | 60,0 | 51,1 | 111,0 | 0,0 |
| Belgium | 4553 | 7,1 | 683 | 14,6 | 60,7 | 49,0 | 135,0 | 0,0 |
| Bulgaria | 3007 | 3,9 | 503 | 14,1 | 39,1 | 28,8 | 110,0 | 0,0 |
| Croatia | 4101 | 3,8 | 525 | 14,6 | 43,1 | 28,3 | 113,5 | 0,0 |
| Cyprus | 3634 | 7,8 | 815 | 17,2 | 60,0 | 47,4 | 123,8 | 0,0 |
| Czechia | 3659 | 4,6 | 481 | 14,0 | 42,3 | 32,2 | 118,9 | 0,0 |
| Denmark | 4464 | 5,4 | 660 | 13,1 | 62,4 | 33,9 | 134,3 | 0,0 |
| Estonia | 5515 | 5,0 | 496 | 14,8 | 43,0 | 33,5 | 123,6 | 0,0 |
| Finland | 5073 | 5,9 | 573 | 13,4 | 60,2 | 56,6 | 130,3 | 0,0 |
| France | 4957 | 5,9 | 762 | 14,0 | 61,6 | 70,1 | 146,0 | 0,0 |
| Germany | 4465 | 5,8 | 736 | 14,3 | 53,8 | 21,1 | 107,8 | 0,0 |
| Greece | 7466 | 5,6 | 708 | 14,2 | 59,8 | 39,6 | 116,2 | 0,0 |
| Hungary | 3725 | 3,9 | 508 | 14,6 | 43,9 | 28,4 | 111,8 | 0,0 |
| Ireland | 5242 | 8,9 | 861 | 18,5 | 50,5 | 75,6 | 166,6 | 0,0 |
| Italy | 4911 | 4,9 | 705 | 15,1 | 60,2 | 32,7 | 119,0 | 0,0 |
| Latvia | 5599 | 5,0 | 527 | 14,3 | 43,1 | 32,2 | 116,7 | 0,0 |
| Lithuania | 5991 | 4,9 | 575 | 14,8 | 43,5 | 30,8 | 110,0 | 0,0 |
| Luxembourg | 3908 | 7,0 | 714 | 16,5 | 60,9 | 46,3 | 127,0 | 0,0 |
| Malta | 3792 | 6,5 | 750 | 14,9 | 59,0 | 41,9 | 110,7 | 0,0 |
| Netherlands | 4547 | 7,6 | 700 | 20,3 | 54,4 | 52,3 | 124,6 | 0,0 |
| Poland | 2407 | 4,3 | 494 | 15,7 | 45,2 | 31,8 | 124,5 | 0,0 |
| Portugal | 6569 | 5,0 | 863 | 14,3 | 59,1 | 36,1 | 111,9 | 0,0 |
| Romania | 3221 | 4,1 | 498 | 14,3 | 50,3 | 31,0 | 124,1 | 0,0 |
| Slovakia | 3269 | 4,6 | 519 | 15,7 | 43,5 | 31,8 | 116,5 | 0,0 |
| Slovenia | 4278 | 4,1 | 502 | 14,2 | 41,9 | 31,3 | 112,2 | 0,0 |
| Spain | 6688 | 5,5 | 630 | 14,1 | 61,2 | 84,6 | 122,1 | 0,0 |
| Sweden | 5238 | 9,6 | 669 | 14,8 | 62,5 | 38,5 | 149,6 | 0,0 |
| UK | 5300 | 10,1 | 648 | 12,8 | 69,2 | 51,8 | 151,2 | 0,0 |
