# Supplementary material for: Mental health status of the European population and its determinants: A cross-national comparison study
Source: Eur Psychiatry. 2025 Apr 24;68(1):e103. doi: 10.1192/j.eurpsy.2025.2449 (PMC12344470; doi:10.1192/j.eurpsy.2025.2449)
Supplement: Lopez-Morinigo et al. supplementary material [file S0924933825024496sup001.zip › Table_S3_YLD_Mort_Suic_Raw.doc]

| **Table S3. Raw data on YLD, mortality and suicide rates** | | | | |
| --- | --- | --- | --- | --- |
|  | **YLDs** | **YLDs <20** | **Mortality rate** | **Suicide rate** |
| Austria | 2048,4 | 1255,4 | 39,9 | 11,68 |
| Belgium | 1997,1 | 727,3 | 47,3 | 15,24 |
| Bulgaria | 2039,0 | 1515,5 | 43,1 | 8,04 |
| Croatia | 1684,4 | 716,3 | 32,7 | 13,19 |
| Cyprus | 2079,4 | 773,7 | 35,3 | 3,45 |
| Czechia | 2203,3 | 1713,2 | 19,1 | 11,53 |
| Denmark | 1941,1 | 1141,3 | 59,0 | 10,01 |
| Estonia | 1826,5 | 746,7 | 13,9 | 16,28 |
| Finland | 1994,7 | 1222,9 | 55,5 | 12,95 |
| France | 2178,3 | 1300,4 | 37,4 | 13,47 |
| Germany | 2073,4 | 1218,0 | 72,1 | 10,3 |
| Greece | 2509,8 | 1392,6 | 25,4 | 4,03 |
| Hungary | 1617,8 | 739,7 | 43,8 | 17,08 |
| Ireland | 2310,7 | 1363,6 | 35,4 | 9,62 |
| Italy | 2150,8 | 1247,6 | 45,7 | 5,61 |
| Latvia | 1871,2 | 753,6 | 42,3 | 15,59 |
| Lithuania | 1988,8 | 816,0 | 15,4 | 21,25 |
| Luxembourg | 2008,4 | 1184,3 | 46,2 | 9,99 |
| Malta | 2050,7 | 1232,4 | 63,0 | 3,98 |
| Netherlands | 2241,5 | 1312,8 | 67,1 | 10,48 |
| Poland | 1842,2 | 869,8 | 43,1 | 11,92 |
| Portugal | 2603,9 | 1558,8 | 62,0 | 8,48 |
| Romania | 2018,9 | 969,7 | 43,1 | 9,04 |
| Slovakia | 2040,4 | 913,8 | 8,9 | 6,88 |
| Slovenia | 1693,4 | 714,5 | 20,2 | 17,01 |
| Spain | 2396,4 | 1442,8 | 43,2 | 7,87 |
| Sweden | 2139,5 | 1183,1 | 63,0 | 11,91 |
| UK | 2095,1 | 1158,8 | 84,0 | 10,5 |
