# Supplementary material for: Mental health status of the European population and its determinants: A cross-national comparison study
Source: Eur Psychiatry. 2025 Apr 24;68(1):e103. doi: 10.1192/j.eurpsy.2025.2449 (PMC12344470; doi:10.1192/j.eurpsy.2025.2449)
Supplement: Lopez-Morinigo et al. supplementary material [file S0924933825024496sup001.zip › Table_S4_Individual_Determinants_Raw.doc]

| **Table S4. Raw data on individual determinants of mental health** | | | | | | | |
| --- | --- | --- | --- | --- | --- | --- | --- |
|  | **Adults** | | | **Under-20** | | | |
|  | **Smoking** | **Alcohol** | **Drugs** | **Smoking** | **Alcohol** | **Bullying** | **Sexual abuse** |
| Austria | 20,6 | 16,4 | 5,5 | 23,0 | 49,0 | 6,8 | 8,3 |
| Belgium | 15,4 | 27,7 | 3,4 | 17,3 | 34,7 | 5,3 | 3,8 |
| Bulgaria | 29,1 | 12,7 | 1,8 | 32,0 | 41,5 | 13,5 | 9,0 |
| Croatia | 22,1 | 16,6 | 4,5 | 29,0 | 45,0 | 7,9 | 2,9 |
| Cyprus | 22,5 | 3,6 | 3,1 | 14,3 | 37,5 | 7,8 | 3,5 |
| Czechia | 16,6 | 21,3 | 5,1 | 23,6 | 38,5 | 8,2 | 8,6 |
| Denmark | 13,9 | 37 | 6,7 | 22,4 | 59,0 | 5,0 | 6,2 |
| Estonia | 17,9 | 14,6 | 5,5 | 19,8 | 32,5 | 8,3 | 4,1 |
| Finland | 12 | 28,3 | 4,3 | 17,3 | 21,5 | 6,2 | 8,0 |
| France | 25,5 | 20,7 | 3,9 | 21,6 | 33,5 | 6,8 | 5,2 |
| Germany | 18,8 | 30,3 | 4 | 19,7 | 54,5 | 6,2 | 14,2 |
| Greece | 24,9 | 6,1 | 1 | 15,0 | 31,5 | 7,6 | 14,6 |
| Hungary | 24,9 | 13,8 | 1,9 | 28,2 | 41,5 | 7,4 | 3,0 |
| Ireland | 16 | 24,8 | 7,8 | 14,3 | 32,5 | 8,7 | 8,9 |
| Italy | 18,8 | 4,3 | 4 | 32,0 | 35,0 | 7,9 | 10,5 |
| Latvia | 22,6 | 14,7 | 2,1 | 22,6 | 35,5 | 11,3 | 3,7 |
| Lithuania | 18,9 | 16,5 | 1,2 | 21,1 | 28,0 | 9,7 | 4,6 |
| Luxembourg | 19,2 | 34,7 | 2,1 | 17,1 | 24,2 | 6,9 | 8,4 |
| Malta | 20,6 | 15,8 | 0,5 | 10,5 | 40,5 | 13,9 | 8,9 |
| Netherlands | 14,4 | 19,4 | 7,9 | 15,0 | 42,5 | 2,3 | 13,7 |
| Poland | 17,1 | 17,5 | 1,4 | 22,0 | 34,0 | 8,3 | 1,4 |
| Portugal | 14,2 | 15 | 0,8 | 14,0 | 24,0 | 5,3 | 13,5 |
| Romania | 18,9 | 36,4 | 0,9 | 31,0 | 37,0 | 11,5 | 3,0 |
| Slovakia | 21 | 12 | 1,6 | 29,0 | 46,5 | 9,2 | 2,8 |
| Slovenia | 17,4 | 22,7 | 2,6 | 19,0 | 40,5 | 6,6 | 3,4 |
| Spain | 19,8 | 6,2 | 6,8 | 21,0 | 34,0 | 5,3 | 8,9 |
| Sweden | 9,5 | 19,4 | 3,4 | 11,0 | 20,0 | 5,6 | 13,0 |
| UK | 14,5 | 29,8 | 3,4 | 16,0 | 32,6 | 10,9 | 11,7 |
