# Supplementary material for: Mental health status of the European population and its determinants: A cross-national comparison study
Source: Eur Psychiatry. 2025 Apr 24;68(1):e103. doi: 10.1192/j.eurpsy.2025.2449 (PMC12344470; doi:10.1192/j.eurpsy.2025.2449)
Supplement: Lopez-Morinigo et al. supplementary material [file S0924933825024496sup001.zip › Table_S6_Environmental_Raw.doc]

| **Table S6. Raw data on environmental determinants of mental health across European countries** | | | | | | | | | |  |
| --- | --- | --- | --- | --- | --- | --- | --- | --- | --- | --- |
|  | **Daylight** | **Temp. Inc.** | **Weather** | **Natural Disasters** | **Ecoanxiety** | **Air Pollution** | **Noise Pollution** | **Traffic** | **Commuting Time** | **Green Infrastructure** |
| Austria | 2048,0 | 2,5 | 1527,0 | 1,14 | 28 | 9,9 | 16,8 | 73,34 | 34,1 | 33,77 |
| Belgium | 1546,0 | 2,8 | 1247,0 | 4,16 | 30 | 9,4 | 14,5 | 147,37 | 38,6 | 36,92 |
| Bulgaria | 2177,0 | 1,8 | 504,0 | 2,15 | 48 | 17 | 8,8 | 111,12 | 40,7 | 36,77 |
| Croatia | 1913,0 | 2,2 | 667,0 | 4,86 | 52 | 15,4 | 8,1 | 115,54 | 33,5 | 46,09 |
| Cyprus | 3314,1 | 1,2 | 565,0 | 2,78 | 57 | 14 | 14 | 108,9 | 28,8 | 23,4 |
| Czechia | 1668,0 | 2,4 | 1479,0 | 1,00 | 24 | 12,5 | 13,3 | 98,72 | 39,3 | 28,6 |
| Denmark | 1912,0 | 2,2 | 1561,0 | 1,03 | 12 | 7,6 | 18,2 | 86,89 | 36,4 | 16,31 |
| Estonia | 1923,0 | 2,0 | 186,0 | 1,82 | 18 | 5,4 | 8 | 87,59 | 30,4 | 43,3 |
| Finland | 1858,0 | 2,1 | 419,0 | 1,30 | 9 | 4,4 | 14,1 | 78,05 | 31,5 | 43,74 |
| France | 1662,0 | 2,9 | 1720,0 | 6,67 | 33 | 8,6 | 20,5 | 149,77 | 35,2 | 20,44 |
| Germany | 1626,0 | 2,6 | 1859,0 | 3,92 | 18 | 9,1 | 21,6 | 102,85 | 35,5 | 43,78 |
| Greece | 2773,0 | 2,1 | 1057,0 | 8,55 | 59 | 14,5 | 20,1 | 157,97 | 23,4 | 10,8 |
| Hungary | 1988,0 | 2,1 | 607,0 | 0,97 | 62 | 14,5 | 9,3 | 141,98 | 43,9 | 26,05 |
| Ireland | 1453,0 | 1,8 | 741,0 | 3,10 | 28 | 7,1 | 10,3 | 159,33 | 37,9 | 20,47 |
| Italy | 2473,0 | 2,1 | 1581,0 | 9,37 | 43 | 15 | 14,3 | 168,63 | 28,0 | 24,08 |
| Latvia | 1754,0 | 2,0 | 453,0 | 2,14 | 26 | 9,1 | 12,5 | 116,6 | 52,4 | 43,03 |
| Lithuania | 1691,0 | 2,0 | 436,0 | 2,24 | 22 | 9,8 | 14,7 | 91,31 | 31,4 | 47,15 |
| Luxembourg | 1634,0 | 2,9 | 1763,0 | 0,52 | 26 | 7,3 | 19,7 | 98,23 | 45,2 | 37,32 |
| Malta | 3054,0 | 2,0 | 129,0 | 0,94 | 63 | 10,1 | 30,8 | 106,46 | 39,0 | 6,58 |
| Netherlands | 1779,0 | 2,0 | 638,0 | 4,04 | 26 | 9,2 | 25,5 | 80,39 | 35,5 | 14,13 |
| Poland | 1997,1 | 2,1 | 432,0 | 4,63 | 56 | 16 | 12,6 | 104,56 | 34,9 | 39,66 |
| Portugal | 2806,0 | 2,3 | 1336,0 | 5,08 | 64 | 8,1 | 25,1 | 128,81 | 25,8 | 16,82 |
| Romania | 2115,0 | 2,3 | 678,0 | 3,19 | 45 | 15,2 | 16,1 | 165,16 | 41,9 | 21,89 |
| Slovakia | 2038,0 | 2,0 | 294,0 | 1,00 | 49 | 14,5 | 9,9 | 104,63 | 28,6 | 36,11 |
| Slovenia | 1974,0 | 2,4 | 1918,0 | 1,44 | 32 | 12,5 | 15 | 98,52 | 28,7 | 50,27 |
| Spain | 2769,0 | 2,5 | 1535,0 | 9,68 | 48 | 10 | 21,9 | 102,25 | 33,3 | 39,43 |
| Sweden | 1803,0 | 2,1 | 409,0 | 2,06 | 21 | 4,8 | 17,3 | 109,41 | 36,4 | 33,89 |
| UK | 1633,0 | 1,9 | 913,5 | 5,78 | 41 | 8,29 | 19,8 | 171,14 | 39,8 | 21 |
