# Supplementary material for: Mental health status of the European population and its determinants: A cross-national comparison study
Source: Eur Psychiatry. 2025 Apr 24;68(1):e103. doi: 10.1192/j.eurpsy.2025.2449 (PMC12344470; doi:10.1192/j.eurpsy.2025.2449)
Supplement: Lopez-Morinigo et al. supplementary material [file S0924933825024496sup001.zip › Table_S7_Enviromental_Determinants_Scores.doc]

| **Table S7. Environmental determinants of mental health across European countries: Headway-transformed KPI scores** | | | | | | | | | | | |
| --- | --- | --- | --- | --- | --- | --- | --- | --- | --- | --- | --- |
|  | **Daylight** | **Temp. Inc.** | **Weather** | **Natural Disasters** | **Ecoanxiety** | **Air Pollution** | **Noise Pollution** | **Traffic** | **Comm. Time** | **Green area** | **TOTAL SCORE** |
| Austria | 3,9 | 3,1 | 3,0 | 9,4 | 6,9 | 6,1 | 6,5 | 10,0 | 6,7 | 6,6 | 6,0 |
| Belgium | 1,4 | 1,5 | 4,4 | 6,4 | 6,6 | 6,4 | 7,4 | 3,2 | 5,3 | 7,2 | 4,5 |
| Bulgaria | 4,5 | 6,8 | 8,1 | 8,4 | 3,6 | 1,0 | 9,7 | 6,5 | 4,6 | 7,2 | 5,9 |
| Croatia | 3,2 | 4,7 | 7,3 | 5,7 | 3,0 | 2,1 | 10,0 | 6,1 | 6,9 | 9,1 | 5,2 |
| Cyprus | 10,0 | 10,0 | 7,8 | 7,8 | 2,1 | 3,1 | 7,6 | 6,7 | 8,3 | 4,5 | 9,6 |
| Czechia | 2,0 | 3,6 | 3,2 | 9,5 | 7,5 | 4,2 | 7,9 | 7,7 | 5,0 | 5,5 | 5,3 |
| Denmark | 3,2 | 4,7 | 2,8 | 9,5 | 9,5 | 7,7 | 6,0 | 8,8 | 6,0 | 3,0 | 5,9 |
| Estonia | 3,3 | 5,8 | 9,7 | 8,7 | 8,5 | 9,3 | 10,0 | 8,7 | 7,8 | 8,6 | 10,0 |
| Finland | 3,0 | 5,2 | 8,5 | 9,2 | 10,0 | 10,0 | 7,6 | 9,6 | 7,5 | 8,7 | 9,6 |
| France | 2,0 | 1,0 | 2,0 | 4,0 | 6,1 | 7,0 | 5,1 | 3,0 | 6,3 | 3,9 | 2,4 |
| Germany | 1,8 | 2,6 | 1,3 | 6,7 | 8,5 | 6,6 | 4,6 | 7,3 | 6,2 | 8,7 | 4,9 |
| Greece | 7,4 | 5,2 | 5,3 | 2,1 | 1,8 | 2,8 | 5,2 | 2,2 | 10,0 | 1,9 | 1,0 |
| Hungary | 3,6 | 5,2 | 7,6 | 9,6 | 1,3 | 2,8 | 9,5 | 3,7 | 3,6 | 5,0 | 4,1 |
| Ireland | 1,0 | 6,8 | 6,9 | 7,5 | 6,9 | 8,1 | 9,1 | 2,1 | 5,5 | 3,9 | 6,1 |
| Italy | 5,9 | 5,2 | 2,7 | 1,3 | 4,4 | 2,4 | 7,5 | 1,2 | 8,6 | 4,6 | 3,7 |
| Latvia | 2,5 | 5,8 | 8,4 | 8,4 | 7,2 | 6,6 | 8,2 | 6,0 | 1,0 | 8,5 | 5,8 |
| Lithuania | 2,2 | 5,8 | 8,5 | 8,3 | 7,9 | 6,1 | 7,4 | 8,3 | 7,5 | 9,4 | 8,1 |
| Luxembourg | 1,9 | 1,0 | 1,8 | 10,0 | 7,2 | 7,9 | 5,4 | 7,7 | 3,2 | 7,3 | 4,9 |
| Malta | 8,7 | 5,8 | 10,0 | 9,6 | 1,2 | 5,9 | 1,0 | 7,0 | 5,2 | 1,0 | 5,3 |
| Netherlands | 2,6 | 5,8 | 7,4 | 6,5 | 7,2 | 6,6 | 3,1 | 9,4 | 6,2 | 2,6 | 6,2 |
| Poland | 3,6 | 5,2 | 8,5 | 6,0 | 2,3 | 1,7 | 8,2 | 7,1 | 6,4 | 7,8 | 5,0 |
| Portugal | 7,5 | 4,2 | 3,9 | 5,5 | 1,0 | 7,4 | 3,3 | 4,9 | 9,3 | 3,1 | 5,0 |
| Romania | 4,2 | 4,2 | 7,2 | 7,4 | 4,1 | 2,3 | 6,8 | 1,6 | 4,2 | 4,2 | 2,3 |
| Slovakia | 3,8 | 5,8 | 9,2 | 9,5 | 3,5 | 2,8 | 9,3 | 7,1 | 8,4 | 7,1 | 6,2 |
| Slovenia | 3,5 | 3,6 | 1,0 | 9,1 | 6,2 | 4,2 | 7,2 | 7,7 | 8,3 | 10,0 | 5,9 |
| Spain | 7,4 | 3,1 | 2,9 | 1,0 | 3,6 | 6,0 | 4,5 | 7,3 | 6,9 | 7,8 | 4,3 |
| Sweden | 2,7 | 5,2 | 8,6 | 8,5 | 8,0 | 9,7 | 6,3 | 6,7 | 6,0 | 6,6 | 7,5 |
| UK | 1,9 | 6,3 | 6,1 | 4,8 | 4,8 | 7,2 | 5,3 | 1,0 | 4,9 | 4,0 | 4,2 |
