# Supplementary material for: Mental health status of the European population and its determinants: A cross-national comparison study
Source: Eur Psychiatry. 2025 Apr 24;68(1):e103. doi: 10.1192/j.eurpsy.2025.2449 (PMC12344470; doi:10.1192/j.eurpsy.2025.2449)
Supplement: Lopez-Morinigo et al. supplementary material [file S0924933825024496sup001.zip › Table_S8_Socieconomic_Raw.doc]

| **Table S8. Raw data on socioeconomic determinants of MH across European countries** | | | | |
| --- | --- | --- | --- | --- |
|  | **Fragile State Index** | **Crime level** | **Overcrowding rate** | **Poor housing** |
| Austria | 24,4 | 37,28 | 15,7 | 9,1 |
| Belgium | 31,4 | 24,76 | 6,2 | 15,7 |
| Bulgaria | 51,8 | 5,49 | 36,2 | 11,0 |
| Croatia | 48,7 | 11,44 | 32,7 | 9,4 |
| Cyprus | 57,0 | 11,60 | 2,2 | 39,1 |
| Czechia | 40,2 | 6,36 | 15,1 | 6,8 |
| Denmark | 17,9 | 33,37 | 9,8 | 16,8 |
| Estonia | 38,6 | 13,69 | 15,7 | 10,2 |
| Finland | 16,0 | 51,50 | 8,4 | 4,5 |
| France | 28,8 | 48,71 | 9,2 | 17,9 |
| Germany | 24,6 | 16,69 | 11,0 | 12,0 |
| Greece | 55,1 | 10,68 | 28,0 | 12,5 |
| Hungary | 48,8 | 3,46 | 17,0 | 20,4 |
| Ireland | 19,5 | 18,28 | 4,3 | 16,6 |
| Italy | 42,6 | 5,46 | 25,1 | 19,6 |
| Latvia | 43,3 | 30,45 | 41,7 | 17,5 |
| Lithuania | 39,4 | 3,96 | 24,6 | 10,9 |
| Luxembourg | 19,5 | 18,84 | 5,7 | 15,4 |
| Malta | 33,0 | 14,82 | 2,8 | 0,1 |
| Netherlands | 21,0 | 12,82 | 2,9 | 14,8 |
| Poland | 45,2 | 5,69 | 35,8 | 6,0 |
| Portugal | 25,7 | 26,16 | 9,4 | 25,2 |
| Romania | 53,0 | 7,99 | 40,5 | 10,0 |
| Slovakia | 37,8 | 5,84 | 31,8 | 4,9 |
| Slovenia | 27,3 | 6,84 | 11,0 | 20,8 |
| Spain | 43,5 | 16,72 | 6,6 | 19,7 |
| Sweden | 20,6 | 72,56 | 17,0 | 7,1 |
| UK | 41,9 | 81,69 | 3,1 | 17,6 |
